# Metal-Free Pyrene-Based Conjugated Microporous Polymer Catalyst Bearing N- and S-Sites for Photoelectrochemical Oxygen Evolution Reaction

**DOI:** 10.3389/fchem.2021.803860

**Published:** 2021-12-24

**Authors:** Sabuj Kanti Das, Sanjib Shyamal, Manisha Das, Saptarsi Mondal, Avik Chowdhury, Debabrata Chakraborty, Ramendra Sundar Dey, Asim Bhaumik

**Affiliations:** ^1^ School of Materials Sciences, Indian Association for the Cultivation of Science, Kolkata, India; ^2^ Institute of Nano Science and Technology, Mohali, India; ^3^ Center for Molecular Spectroscopy and Dynamics, Institute of Basic Science (IBS), Seoul, South Korea; ^4^ Department of Chemistry, Korea University, Seoul, South Korea

**Keywords:** metal-free heterogeneous photoelectrocatalyst, conjugated microporous polymer, high surface area, OER, water splitting

## Abstract

The development of an efficient, sustainable, and inexpensive metal-free catalyst for oxygen evolution reaction (OER) *via* photoelectrochemical water splitting is very demanding for energy conversion processes such as green fuel generators, fuel cells, and metal-air batteries. Herein, we have developed a metal-free pyrene-based nitrogen and sulfur containing conjugated microporous polymer having a high Brunauer-Emmett-Teller surface area (761 m^2^ g^−1^) and a low bandgap of 2.09 eV for oxygen evolution reaction (OER) in alkaline solution. The *π*-conjugated as-synthesized porous organic material (PBTDZ) has been characterized by Fourier transform infrared spectroscopy (FT-IR), solid-state ^13^C (cross-polarization magic angle spinning-nuclear magnetic resonance) CP-MAS NMR, N_2_ adsorption/desorption analysis, field-emission scanning electron microscope (FESEM), high-resolution transmission electron microscopy (HRTEM), X-ray photoelectron spectroscopy (XPS) and thermogravimetric analysis (TGA) experiments. The material acts as an efficient catalyst for photoelectrochemical OER with a current density of 80 mA/cm^2^ at 0.8 V vs. Ag/AgCl and delivered 104 µmol of oxygen in a 2 h run. The presence of low bandgap energy, *π*-conjugated conducting polymeric skeleton bearing donor heteroatoms (N and S), and higher specific surface area associated with inherent microporosity are responsible for this admirable photoelectrocatalytic activity of PBTDZ catalyst.

## Introduction

The massive rise of the global population over recent decades is causing a huge increase in the demand for energy for society (Adam, 2021). Thus, the limited stock of fossil fuels (Bebbington et al., 2020) will cause a serious energy crisis (Manieniyan et al., 2009) in near future. Thus, finding a sustainable alternative energy resource became one of the major modern concerns. Moreover, in the last 2 years world has been facing a pandemic due to COVID-19, and a sustainable supply of oxygen for COVID-affected patients is essential for their survival. Therefore, oxygen generation in a sustainable way is highly necessary to address both the energy and health crises. In this context the water splitting (Maeda and Domen, 2010; Ifkovits et al., 2021) reaction is the most promising solution considering the abundance of water in nature. The overall water splitting reaction proceeds through two half-cell reactions (Zhao and Liu, 2014): hydrogen evolution reaction (HER) and oxygen evolution reaction (OER). Hydrogen evolution reaction proceeds via two-electron transfer, while the oxygen evolution proceeds via four electrons transfer process (Suen et al., 2017). As a consequence, its ubiquitously evident that the oxygen evolution reaction is immensely more kinetically adverse than the hydrogen evolution pathway. So, OER demands low overpotential (Peng et al., 2018) for overcoming the high kinetic energy barrier associated with multiple electron transfer.

The water oxidation process plays a significant role in many energy conversion devices, like fuel cells (Dresp et al., 2016), water electrolyzers, and rechargeable metal-air batteries (Zhang et al., 2020). Among the several strategies, photoelectrochemical water splitting is considered to be crucial for the generation of alternative energy resources by utilizing solar energy. After the successful demonstration by Fujishima and Honda, and Finegold and Cude (1972) with TiO_2_ photoelectrode displaying water splitting under UV-Vis light, a massive interest has developed in searching for suitable photocatalysts for furnishing water splitting. So far a wide variety of materials have been explored as a catalyst for OER under visible and ultraviolet irradiation. Several nanostructured metal oxides, mixed metal oxides, perovskite, heteroelement doped porous carbons (Liu et al., 2020), etc. showed interesting results. Metal oxides like RuO_2_ and IrO_2_ (Antolini, 2014; Moradi and Dehghanian, 2014) showed good catalytic activity with 300 mV overpotential at 10 mA/cm^2^ current density. Bismuth vanadate (BiVO_4_) (Jo et al., 2012) has attracted huge attention in this context by virtue of its narrow bandgap (2.4 eV). As per the literature, cobalt oxide (Lv et al., 2014), nickel-cobalt oxide (Chen et al., 2017), porous mixed metal phosphate (Chatterjee et al., 2020), W-doped BiVO_4_ (Yuan et al., 2020), heterojunction photoanodes (Chatterjee et al., 2021), etc. were found to display significant OER activity under visible light radiation. Interestingly, in one of our recently published works, we found mixed metal phosphonate open framework material NiWPPA-4 (Chakraborty et al., 2020) as an effective OER photocatalyst under visible light irradiation. Metal-porphyrins also displayed good light-harvesting properties under visible light irradiation (Di Carlo et al., 2019). Although, several benchmark photocatalysts have been discovered over the years, their serious disadvantages like high overpotential, low current density, along high cost are largely motivating the scientific community to find a sustainable and cost-effective OER catalyst with high efficiency and durability.

**Figure:**
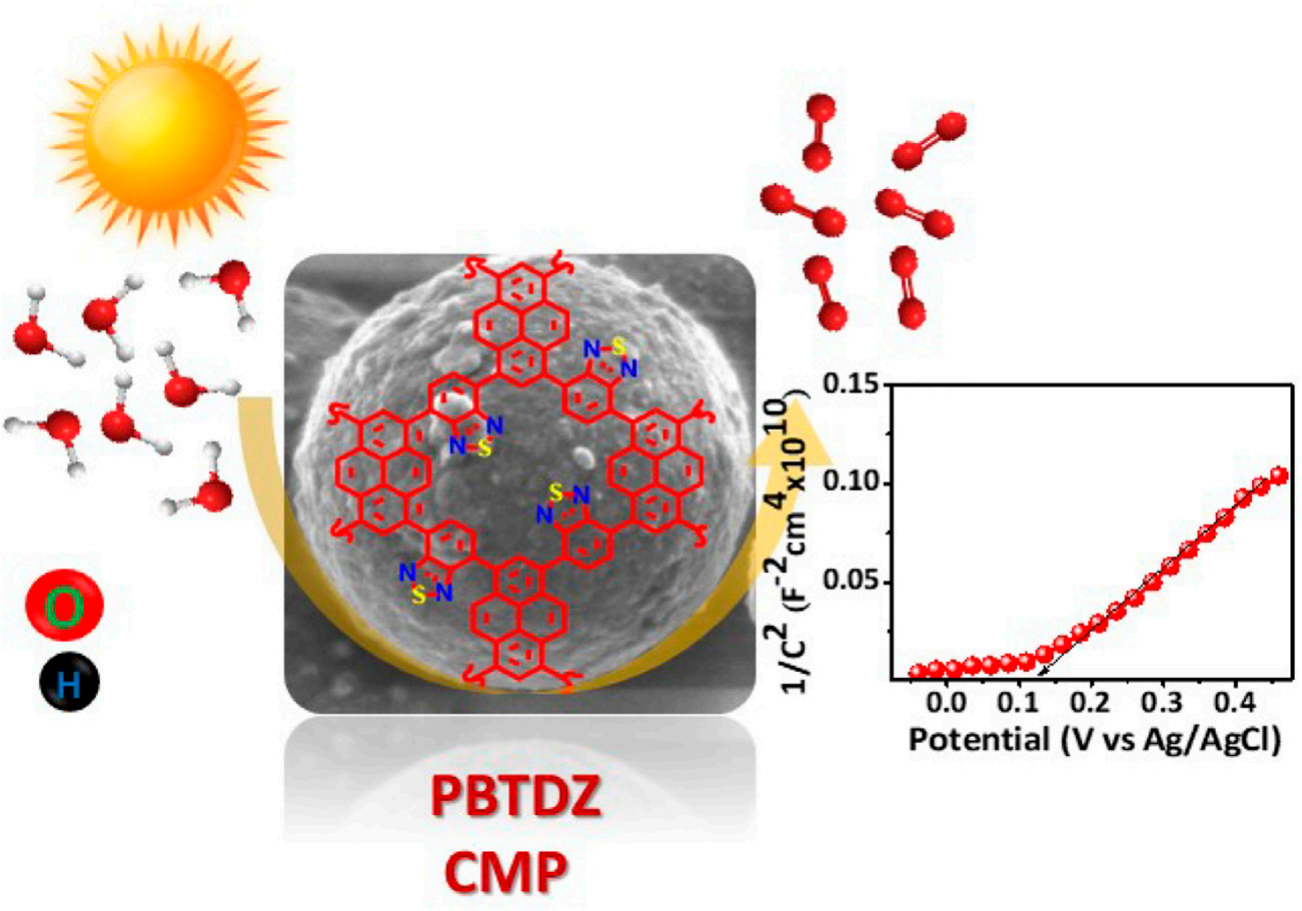


**Scheme 1:**
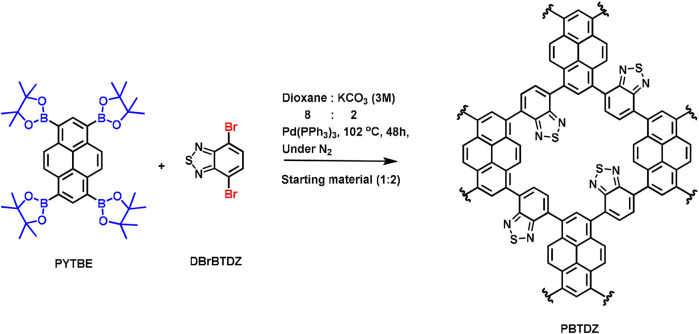
Synthesis of PBTDZ via Suzuki C-C cross-coupling reaction.

From the last few decades, porous nanomaterials have attracted huge attention in addressing some of the major commercial/household problems. Their surface-active porous architecture makes them promising material within a certain domain of applications such as gas storage (Deegan et al., 2021), sensing (Ramírez-González et al., 2020), and separation (Hiraide et al., 2020), as well as in biomedical research. Additionally, the ease of fabrication of organic functional groups at the pore surface and high specific surface area make them desirable materials for various energy harvesting applications. Several members of this family of materialssuch as metal-organic framework (MOF) (Ma et al., 2020), metalized/metal free covalent organic framework (COF) (Lin et al., 2018; Hosokawa et al., 2021), and porous organic polymer (POP) (Wang et al., 2020), have already been utilized as efficient OER photo-electrocatalysts. In this context, it is pertinent to mention that metal-base catalysis is associated with some major drawbacks, such as significant metal leaching and a complicated multi-step synthetic route, which creates serious obstacles towards OER performance. MOFs and COFs materials also draw significant attention in the catalysis field due to their crystalline nature, possibilities in structural engineering, and high specific surface area with tunable porosity. But low solvent stability and lack of large-scale synthesis capability of those materials are the major drawbacks for their applications in heterogeneous catalysis. Thus, considerable attention has been paid to the fabrication of metal-free photo-electrocatalysts (Tang et al., 2017; Tang et al., 2018; Tang and Zhang, 2017; Wang et al., 2019). Interestingly, conjugated microporous polymers (CMPs) have become of great interest in optoelectronics due to their large scale facile synthesis, feasibility for metal grafting or heteroatom doping, post-synthetic modification, highly porous surface, and low band gaps (Cooper, 2009) (Lee and Cooper, 2020). Due to the wide scopes in bandgap tenability, surface functionalization, and structural sustainability, CMPs are being considered as a very useful class of efficient electro/photo-electrocatalysts (Zhang et al., 2019; Taylor et al., 2020). However, only a few CMPs have been studied as photo-electrocatalytic OER catalysts so far (Jayanthi et al., 2017). Heteroatoms like N, S, B, P, and Si, entrenched in carbon sheets, modify the electronic structure of the carbon frameworks by tailoring the Fermi energy levels and tuning the local electronegativity. Heteroatom containing extended *π*-conjugated organic polymer materials have been already established as an efficient candidate towards electro/photo/photo-electro catalytic activity in water oxidation (Xu et al., 2016; Xie et al., 2017; Priyadarsini and Mallik, 2021). Recently, S and N embodied porous organic frameworks have been explored in detail towards catalytic activity. Conclusively, the N and S incorporated porous materials with extended *π*-conjugation tunes the HOMO and LUMO bandgap of the catalyst, which plays a crucial role in the activity towards photoelectrochemical OER. Yu et al. have reported N and S Co-doped graphite foam as a self-supported metal-free electrocatalytic electrode material for water oxidation (Yu et al., 2016). A very low overpotential of 0.380 V with a current density of 10 mA cm^−2^ has been achieved. Hou et al. have likewise reported on S, N co-doped graphene quantum dots (S,N-GQDs) and explored the enhanced activity in photo-catalytic water oxidation (Xie et al., 2017).

Inspired by the admirable activity and versatility of such a catalyst, herein, we report a new *π*-conjugated CMP as a metal-free heterogeneous photocatalyst for OER. In this work we have synthesized N and S-containing pyrene based conjugated microporous polymer PBTDZ through Suzuki C-C cross-coupling reaction (Scheme 1) between 1,3,6,8-tetrakis (4,4,5,5-tetramethyl-1,3,2-dioxaborolan-2-yl) pyrene (PYTBE) and 4, 7-dibromo-2,1,3-benzothiadiazole (DBrTDZ). PBTDZ possesses a highly specific surface area with a low bandgap, and it shows excellent activity and recyclability in the photoelectrochemical water oxidation reaction.

## Experimental Section

### Materials

All reagents were used in the synthesis was utilized without further purification. Tetrakistriphenylphosphine)palladium (0) (99%) [1,1′bis (diphenylphosphino) ferrocene]dichloro palladium (II)], 2,1,3-benzothiadiazole (98%), were received from sigma Aldrich. Bis (PINACOLATO) DIBORON (>98%), K_2_CO_3_, HBr and Br_2_ solutions were purchased from Spectrochem, India. Other solvents were procured from local commercial sources. 1,3,6,8-tetrakis (4,4,5,5-tetramethyl-1,3,2-dioxaborolan-2-yl)pyrene (PYTBE) and 4, 7-Dibromo-2,1,3-benzothiadiazole (DBrTDZ) were synthesized following the previously reported procedure (Bhunia et al., 2017).

### Instrumentation

^1^H and ^13^C NMR spectra were acquired by using Bruker DPX-300 NMR spectrometer. Perkin Elmer 2,400 Series II CHN analyzer was used for the estimation of carbon, hydrogen, and nitrogen contents in PBTDZ catalyst. X-ray powder diffraction patterns of the samples were obtained by using a PANAlytical X’Pert PRO diffractometer with Cu Kα (*λ* = 0.15406 nm) radiation. Autosorb 1 (Quantachrome Corporation, United States) analyzer was used to volumetric nitrogen adsorption/desorption, Brunauer-Emmett-Teller (BET) specific surface area, pore-volume, and micropore size analysis at 77 K. Prior to adsorption measurement, the samples were outgassed in vacuum at 150°C for 10 h. NLDFT pore-size distribution was obtained from the adsorption/desorption isotherms by using the carbon/slit-cylindrical pore model. Whereas, the ^13^C cross-polarization magic angle spinning (CP-MAS) NMR spectrum was acquired at 8 kHz mass frequency by using a 500 MHz Bruker Avance II spectrometer. Thermogravimetric analysis (TGA) was carried out by using thermal analyzer TA-SDT Q-600 TA Instruments. A Hitachi S-5200 field-emission scanning electron microscope (FESEM) was used for the evaluation of the morphology of the as-synthesized material. Transmission electron microscopy (TEM) images were obtained by a JEOL JEM 2010 TEM operating at 100 kV. For the TEM analysis, the samples were prepared by dropping a colloidal solution onto the carbon-coated copper grids followed by drying under a high vacuum. The ^1^H and ^13^C NMR spectra were obtained from Bruker ADVANCE III-400 MHz spectrometer. ^1^H NMR spectra were collected at 400 MHz with chemical shift referenced to the residual peak in CDCl_3_ *δ*: H 7.26 ppm. Multiplicities are written as s (singlet), d (doublet), t (triplet), m (multiplet), and br (broad).

Thin films of the as-prepared metal-free PBTDZ materials are developed on indium tin oxide (ITO) coated glass substrate. The material ink was prepared by dispersing 5 mg synthesized materials in 200 µl of isopropanol and 20 µl of Nafion (5%) solution then the mixture was sonicated for 30 min. Different amounts (2, 5, 10, 20 µl) of prepared ink were drop cast on ITO substrate and dried under vacuum at 60 ^o^C for 2 h. All the electrochemical measurements were performed on a Biologic SP-300, electrochemical workstation with a three-electrode system at room temperature. The three electrode set-up consists of platinum wire as counter, saturated silver/silver chloride electrode (Ag/AgCl) as reference electrode and a PBTDZ coated indium tin oxide (ITO) as the working electrode. The photoelectrodes were exposed to the electrolyte through an O-ring with a working surface area of 0.27 cm^2^. The current-potential measurement was done through linear sweep voltammetry (LSV) (scan rate of 10 mV/s) in 1 (M) KOH electrolyte solution under chopped light illumination of 100 mW/cm^2^ irradiating from a 300 W Xenon arc lamp source. Stability of the prepared thin films was performed through chronoamperometric measurement at a constantly applied bias of 0.6 V vs. Ag/AgCl under continuous illumination for 10 min. Electrochemical impedance spectroscopy (EIS) measurements were carried out at a particular frequency by varying the applied potential (Mott-Schottky) in dark conditions in the above-mentioned electrolyte and the Nyquist experiment has been done at a particular potential (0.6 V vs. Ag/AgCl) in the frequency range of 100 kHz to 20 mHz under dark and illuminated circumstances with an AC amplitude of 10 mV. Gas chromatography (GC) measurement was carried out by employing 1 cm × 1 cm of PBTDZ coated thin film at a fixed applied bias 0.6 V vs. Ag/AgCl in 1 M KOH electrolyte solution under continuous illumination of 100 mW/cm^2^ lamp source in a closed glass reactor connected with a Thermo Scientific (Trace 1,110) gas chromatography using thermal conductivity detector (TCD). Thus, the area under the peak corresponds to the amount of gas generated and this is used for quantitative estimation of oxygen produced.

### Synthesis of 1,3,6,8-Tetrakis (4,4,5,5-Tetramethyl-1,3,2-Dioxaborolan-2-yl) Pyrene (PYTBE)

1.5 g of 1,3,6,8-tetrabromopyrene (2.9 mmol), bis(pinacolato)-diboron (4.4 g, 17.35 mmol), Pd (dppf) Cl_2_ (0.175 g, 0.25 mmol) and potassium acetate (1.75 g, 17.85 mmol) in 15 mL anhydrous dimethyl sulfoxide (DMSO) were charged in a Schlenk tube. The mixture was then purged with N_2_ gas three times followed by heating at 90^°^C for 48 h under constant stirring (Bhunia et al., 2017). Then the reaction mixture was allowed to cooled to room temperature followed by filtration using dichloromethane (DCM). The crude product (yellow solid) was extracted by solvent evaporation technique. It was further purified with dichloromethane/toluene as an eluent using a flash column chromatography separation technique on silica gel (60–120 mesh). The resulting tetraborylated product was received as a grey solid (1.5 g, yield 76%). ^1^H NMR spectra and NMR details of PYTBE are provided in ESI, Supplementary Figure S1.

### Synthesis of 4, 7-Dibromo-2, 1, 3-Benzothiadiazole (DBrBTDZ)

For the synthesis of 4,7-dibromo-2,1,3-benzothiadiazole (Zahara et al., 2020), the bromine (100.0 g, 0.63 mol) in HBr (150.0 mL, 48%) was dropwise added into a solution of benzothiadiazole (30.0 g, 0.22 mol) in HBr (300.0 mL, 48%) at room temperature. After the addition of bromine, the resulting milky reaction mixture was heated at 25^°^C at reflux for 7 h. After the cooling of the mixture at room temperature, a saturated solution of Na_2_SO_3_ (300.0 mL) was added dropwise for the removal of excess bromine. After the filtration in a vacuum and exhaustively washing with water and methanol, a light-yellow solid was obtained, which was then dried under vacuum to a final product yield of 96% (62.0 g). ^1^H NMR and ^13^C NMR spectra and NMR details of DBrBTDZ are provided in ESI, Supplementary Figure S2 and Supplementary Figure S3.

### Synthesis of PBTDZ

A Schlenk flask was charged with 1,3,6,8-tetrakis (4,4,5,5-tetramethyl-1,3,2-dioxaborolan-2-yl) pyrene (PYTBE) (1.412 g, 2 mmol) and 4, 7-dibromo-2,1,3-benzothiadiazole (DBrBTDZ) (1.176 g, 4 mmol), 1,4 dioxane (24 mL), and an aqueous solution of 6 mL (3M) K_2_CO_3_ and Pd(PPh_3_)_4_ (17 mg). After that, the mixture was allowed to be backfilled with nitrogen gas (N_2_) through freeze pump thaw and the process was repeated three times. After that, it was heated to 102°C for 48 h under inert conditions. After this, the whole mixture was cooled down to room temperature, poured into a water-methanol mixture, and then filtration was done to collect the precipitate followed up by washing it with plenty of H_2_O and with MeOH, THF, CH_2_Cl_2_, and acetone (CH_3_COCH_3_) to eliminate the remaining entrapped impurities. The process of purification of the polymer was furthermore carried out by Soxhlet extraction method with MeOH:THF (1:1) for 2 days and then the resultant product was dried under reduced pressure. Finally, the isolated yield of the final product namely PBTDZ was calculated and found to be 76%.

## Results and Discussion

### Physical Characterizations

The *π*-conjugated microporous polymer (PBTDZ) was successfully synthesized through Suzuki C-C cross-coupling reaction between tetrapodal (PYTBE) and bipodal (DBrBTDZ). The CMP PBTDZ material was characterized initially by FTIR and solid-state.

**FIGURE 1:**
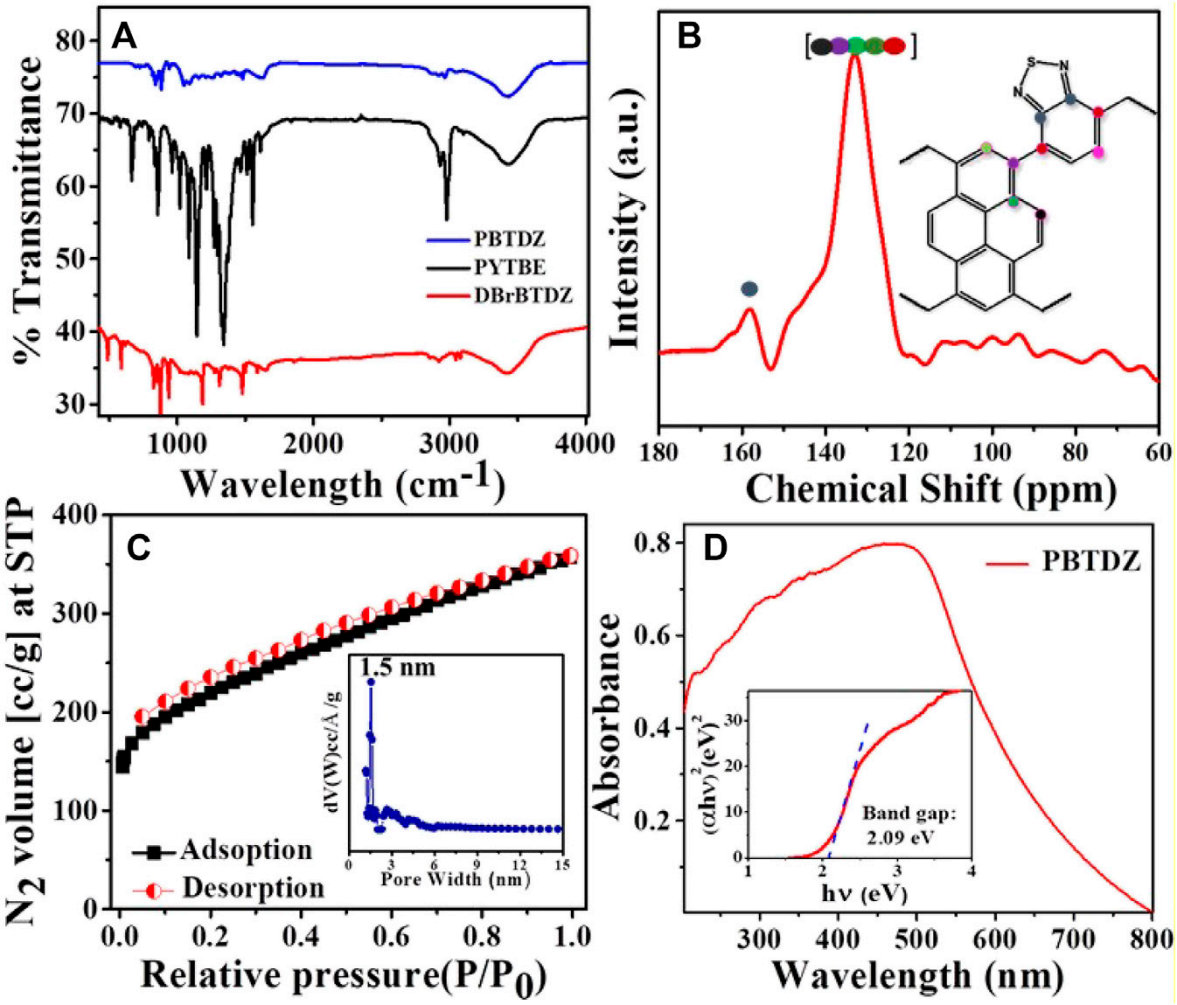
**(A)** FTIR spectra of PBTDZ, PYTBE, DBrBTDZ **(B)** Solid state ^13^C NMR of PBTDZ **(C)** N_2_ adsorption/desorption isotherm of PBTDZ at 77 K **(D)** UV-vis spectra of PBTDZ.

NMR analysis. Bonding connectivity of the materials was confirmed from FTIR spectra as shown in Figure 1A, recorded in the range of 400–4,000 cm^−1^. The presence of the FTIR band at 425 cm^−1^ in DBrBTDZ monomer unit and attenuation of that peak in as-synthesized PBTDZ material is the clear indication of the polymerization process through the C-C coupling reaction. Moreover, the successful synthesis of the material was also confirmed from the distinctive IR stretching band of the aromatic ring and -C=N bond of DBrBTDZ, which appeared at 1,480 and 1,623 cm^−1^, respectively (Zhang et al., 2019). The peak at 2,965 cm^−1^ could be attributed to the stretching frequency of aromatic C-H bonds of PBTDZ. Post experimental FTIR has been done with the recovered sample (ESI, Supplementary Figure S4). The FTIR plot was very similar to the FTIR of as-synthesized material, which indicates the structural integrity and robust nature of the catalyst. For further confirmation of PBTDZ synthesis, solid-state ^13^C NMR data has been recorded (Figure 1B) in which the broad peaks which appeared from 120 to 150 ppm suggested the existence of aromatic C atoms of pyrene as well as benzothiadiazole moieties. The sharp peaks at 158 ppm appeared due to the presence *sp* ^*2*^ hybridized “C” attached with “N” (–C=N) in benzothiadiazole, which confirmed the presence of benzothiadiazole in the polymeric network. The crystallinity of the CMP was analyzed.

Through powder x-ray diffractometry (PXRD) analysis in the two theta range of range 5–60° degrees (ESI, Supplementary Figure S5). A broad peak between 15 and 30°degrees appears, which could be attributed to the amorphous nature of the PBTDZ catalyst (Das et al., 2019). The thermogravimetric analysis (TGA) of the CMP was conducted for understanding the thermal stability of the material. TGA profile diagram shows that the material is thermally stable up to 335°C, which indicates the structural integrity of PBTDZ (ESI, Supplementary Figure S6). N_2_ adsorption/desorption analysis of the CMP was conducted at −196°C (liquid N_2_ temperature, Figure 1C) for understanding the porosity and surface area of the material. As seen from this isotherm that PBTDZ displayed large capillary uptake at low P/P_0_ followed by a steady increase in N_2_ uptake suggesting the presence of mostly large micropores to moderate mesopores (Kundu and Bhaumik, 2015). The Brunauer-Emmett-Teller (BET) surface area and peak pore size obtained from this isotherm were 761 m^2^g^−1^ and 1.5 nm, respectively. To check the solvent stability and sustainability of the photoelectrocatalyst PBTDZ, N_2_ adsorption/desorption experiment has been carried out with the material after immerging it for 5 days at 1 (M) KOH solution (ESI, Supplementary Figure S7). The observed BET surface area of PBTDZ after this treatment was 742 m^2^ g^−1^ with a peak pore size distribution at 1.5 nm. These surface area and porosity analyses also confirmed the stable nature of the cross-linked extended polymer network throughout the material. For the photoelectrochemical process, the bandgap of the material plays a crucial role in the overall efficiency of the catalyst. From solid-state UV-vis spectrum (Figure 1D) the measured band gap of PBTDZ was found to be 2.09 eV. This low bandgap of the material is very much required for superior activity in the photoelectrochemical OER process.

**FIGURE 2:**
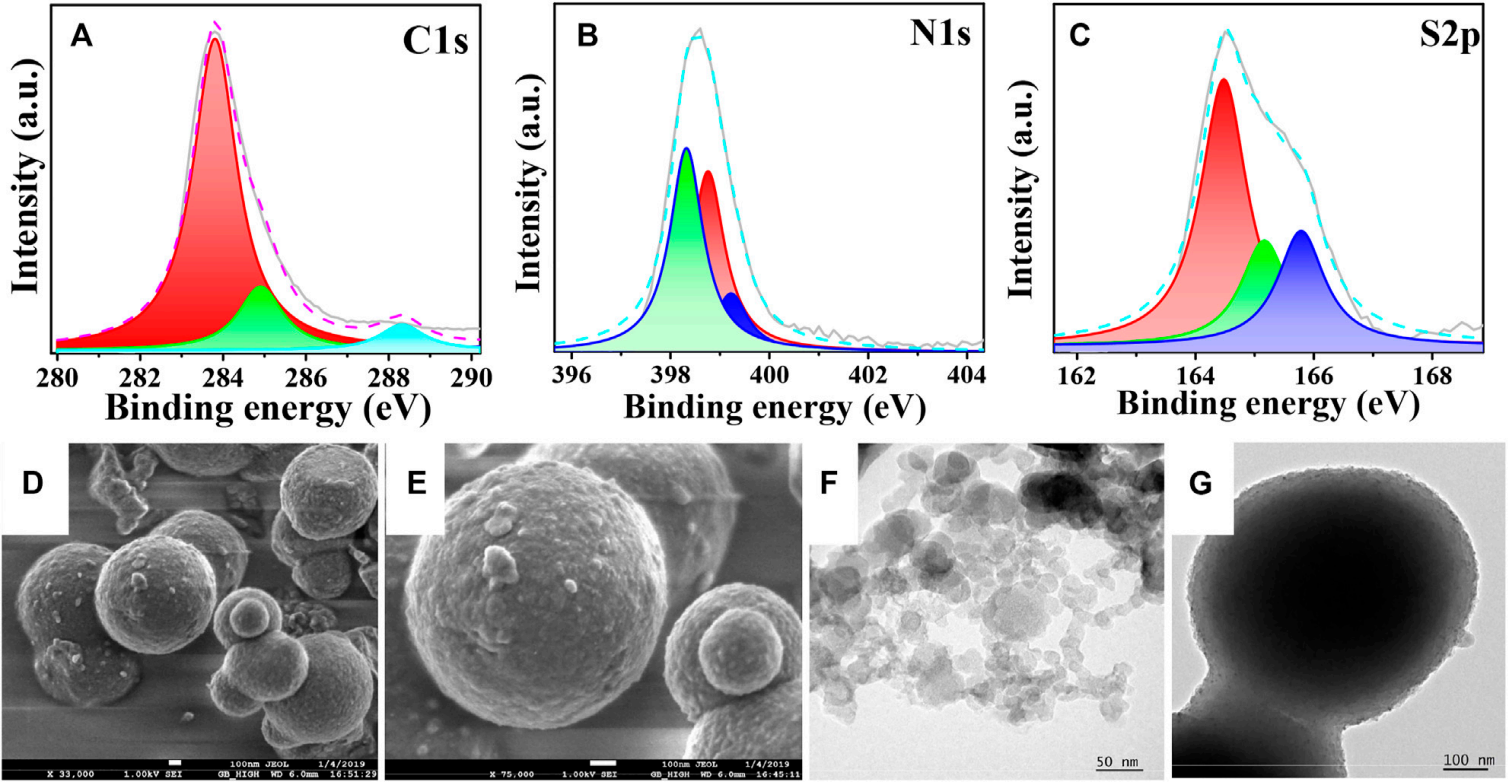
XPS spectra of PBTDZ **(A)** C1s **(B)** N1s **(C)** S2p; FESEM images of PBTDZ **(D,E)**. TEM images of PBTDZ **(F,G)**.

For the interpretation of the local environment of atoms present in the sample, an X-ray photoelectron spectroscopic (XPS) analysis was performed. Figure 2A discloses the binding energy of C1s, where the peak at 283.9 eV appeared due to the existence of the C-C bond (Das et al., 2019) (Kanti Das et al., 2018). The evidence for the peak of the C-N bond was found at 285 eV, and the peak at 288.3 eV can be attributed to the presence of C=O. As depicted in Figure 2B, the binding spectra of N1s shows the peaks at 398.3, 398.7, and 399.1 eV for C-N/C=C, C=N, and N-O, respectively. The binding spectra of S 2p_1/2_ and 2p_3/2_ showed three distinct peaks as shown in Figure 2C. The peak at 165.8 eV was attributed to the occurrence of oxygen-sulfur species, which might have arisen due to air oxidation of sulfur species. The peaks at around 164.3 and 165.1 eV are attributed to the N-S and -C-S-C- groups present in PBTDZ material. Thus, the structure of the heteroatom-containing covalently bonded polymeric framework was confirmed from this XPS analysis. The Full scan XPS profile is shown in ESI, Supplementary Figure S8, and no peaks for Pd 3d3/2 and 3d5/2 were observed at 330–345 eV region indicating that there is no residual Pd present in the metal-free organo-catalyst (Wang et al., 2017). Whereas, the morphological study of the material has been performed by using field-emission scanning electron microscopy (FESEM) and high-resolution transmission electron microscopy (HRTEM) analyses. Because of the self-assembled agglomeration of the material, small spherical particles having a diameter of 10–12 nm combine to form large spherical particles with a diameter of 400 nm, as seen in Figure 2 d-g. To investigate the morphological sustainability and integrity of the materials, post photoelectrocatalytic experimental FESEM and HRTEM analyses have been carried out with the recovered sample (ESI, Supplementary Figure S9), where no noticeable changes with electronic microscopic images of the as-synthesized material were observed.

**FIGURE 3:**
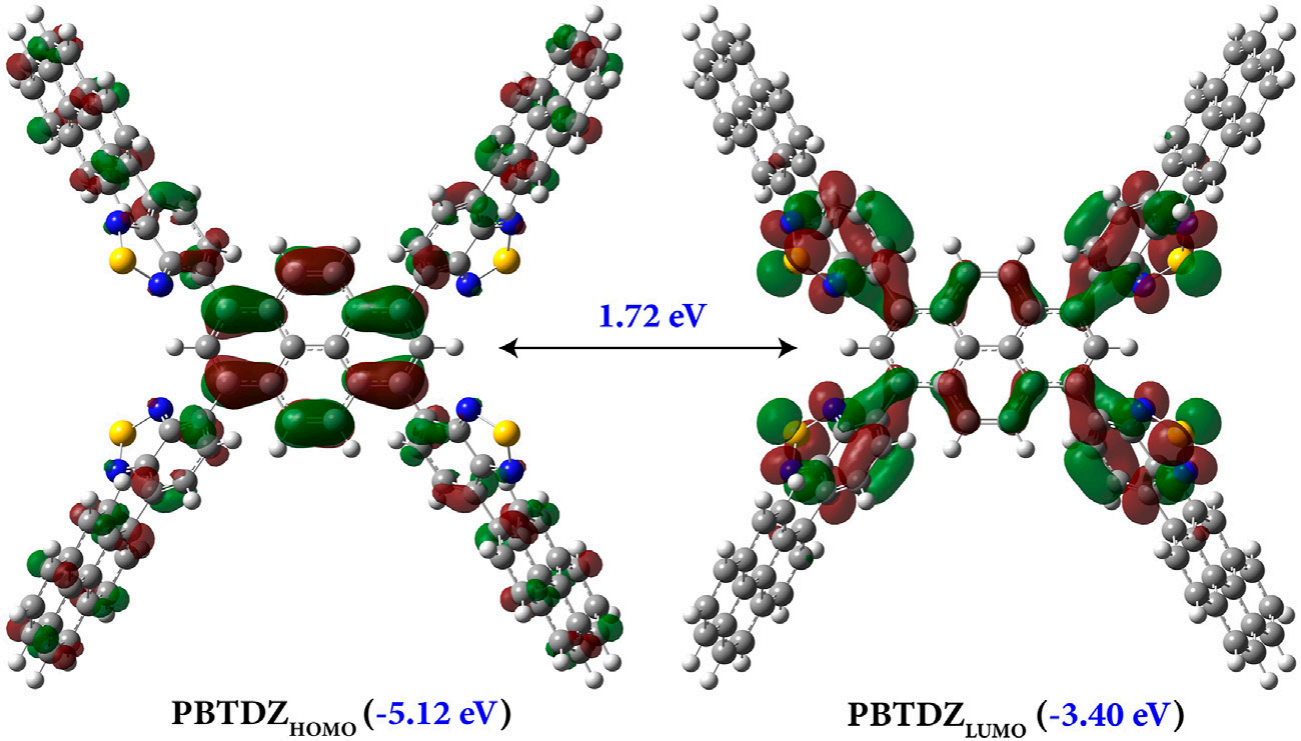
Theoretically generated HOMO and LUMO of PBTDZ using the HCTH407/6-311++G (2DF, 2PD) level of theory.

From solid-state UV-vis spectra analysis, the calculated bandgap of the material was about 2.0 eV, which is very suitable for photoelectron catalysis reaction. To investigate the theoretical bandgap, we performed a quantum chemical calculation to get the HOMO-LUMO energy level of the optimized structure of PBTDZ as shown in Figure 3. The PBTDZ was optimized using ab-initio quantum chemical calculation at RB3LYP-D3/6-311+G (D, P) level of theory using Gaussian 16 suite (Frisch et al., 2016). The frozen core approximation and very tight convergence criterion were employed during geometry optimization. Harmonic frequency calculation was performed to ensure that the structure is at local minima. The highest occupied molecular orbital (HOMO) and lowest unoccupied molecular orbital (LUMO) were generated from the optimized geometry. The energy level corresponding to HOMO is ∼ −5.12 eV, and LUMO was −3.40 eV, accounting for 1.72 eV of the energy gap between HOMO and LUMO, which is in good agreement with the experimental data. In the polymeric structure, the benzothiodiazole unit was connected with pyrene moiety through free rotation and allowed -C-C- covalent bonding interaction. So, the material was allowed to achieve geometrical strain-free orientation through **>** C-C**<** bond rotation as well as stabilized by *π*-*π* interactions which might be the cause of the low bandgap of the material. The HOMO distribution is more broadly scattered along with the whole material and the pyrene ring, the LUMO distribution is more concentrated on the pyrene and benzothiodiazole moiety only, which shows increased planarity between them.

### Photoelectrochemical Water Oxidation

**FIGURE 4:**
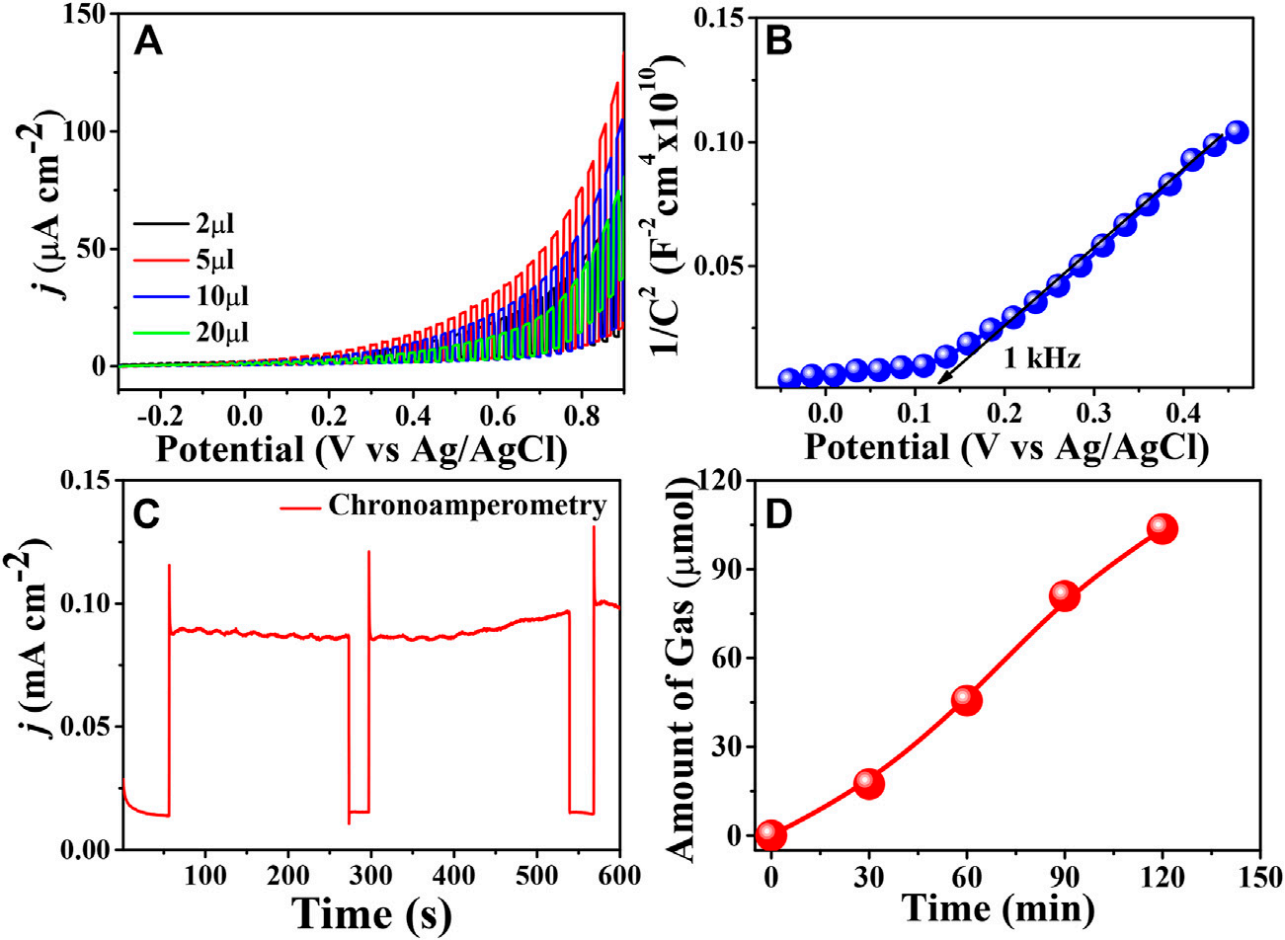
**(A)** Current-potential plot (LSV) of the prepared metal free organic based moiety with different amounts of drop-casted electrodes in 1 M KOH solution **(B)** Mott-Schottky plot of the prepared metal free organic based moiety with 5 µl of drop-casted electrodes **(C)** chronoamperometric plot at fixed bias of 0.6 V vs. Ag/AgCl for 10 min **(D)** GC plot of PBTDZ with 5 µl of drop-casted electrodes (1 cm × 1 cm) in 1 M KOH solution at fixed potential 0.6 V vs. Ag/AgCl under continuous illumination of 100 mW/cm^2^.

The efficacy of the metal-free organic PBTDZ nanoporous material was tested through LSV measurement in 1 M KOH solution under chopped light illumination of 100 mW/cm^2^ in the potential region of −0.3–0.9 V vs. Ag/AgCl shown in Figure 4A. From the graph, it is evident that the maximum photocurrent density of 80 μA/cm^2^ was obtained for 5 µl of catalyst (which was obtained by varying mass loading) towards water oxidation. The low value of photo-electro current for 20 and 10 µL modified electrodes was due to the formation of a thicker film over ITO resulting in the sluggish kinetics of the charge carrier and recombination. Therefore, the synthesized metal-free organic-based material PBTDZ proved to be an effective water oxidation reaction pathway under illumination conditions. Figure 4B represents the Mott-Schottky plot, where the electrodes were scanned within the potential range of −0.1–0.5 V vs. Ag/AgCl at a particular frequency of 1 kHz. From the plot, it is.

Evidence that the prepared materials are n-type in nature with a positive slope is further confirmed from the positive photocurrent obtained in LSV measurements. The applicability of the as-prepared material was tested through stability measurements with a chronoamperometric technique at a fixed applied bias of 0.6 V vs. Ag/AgCl as shown in Figure 4C. It can be observed from the figure that the initial photocurrent of 75 μA/cm^2^ is maintained after 10 min of catalytic reaction. This suggests the photoelectrochemical stability of the synthesized materials towards water oxidation reaction. The electrochemical parameters that affect the water oxidation activity of the PBTDZ were tested through Electrochemical impedance spectroscopy (EIS) measurements (Sarkar et al., 2020). The water oxidation kinetics of the photoelectrodes was further tested through EIS measurements (Nyquist plot) and presented in ESI, Supplementary Figure S10. The diameter of the semi-circles represents the charge transfer resistance (R_ct_) at the electrode-electrolyte interface. The lower the diameter, the more facile the charge transfer reaction is. From the figure, it is evident that upon illumination, the diameter of the semicircles reduced significantly, showing lowering the R_ct_ at the interface, which confirms the photo absorptive nature of the CMP. The R_ct_ value under illumination is 4,538 Ω whereas for dark conditions this value was quite high at 11,537 Ω (Supplementary Figure S10) suggesting the high potential of PBTDZ in photocatalysts. Further confirmation of the photoelectrocatalytic water oxidation reaction and for measuring the amount of gas generated, a gas chromatography (GC) was used. Quantitative oxygen evolution is shown in Figure 4D. The GC analysis confirms that the produced gas is oxygen, which was obtained from the water via oxidation under alkaline conditions. The amount of the gas evolved was 104 µmol after 2 h of photoelectrochemical measurements from 1 M KOH aqueous solution under continuous illumination. The admirable photo-electrochemical activity of the metal-free catalyst is evidenced from the generation of active sites due to the presence of S and N moieties in the porous *π*-conjugated polymer framework. Additionally, the theoretical, as well as experimental studies, also suggested N and S containing carbon atoms resulted in the redeployment of spin and charge densities (Liang et al., 2012).

## Conclusion

An efficient and cost-effective metal-free conjugated microporous polymer PBTDZ was synthesized via Suzuki C-C cross-coupling reaction between a tetraboronate ester and 4,7-dibromo-2,1,3-benzothiadiazole. The physical characterizations of the PBTDZ material confirmed the extended *π*-conjugation of sp^2^ hybridized carbon atoms with N-S heteroatom moieties. The material displayed visible light-induced photoelectrocatalytic activity with n-type semiconducting characteristics, exhibiting the positive photocurrent at various potentials. The CMP material played a significant role as a photo-electrocatalyst for OER in 1M KOH exhibiting a current density of 80 mA/cm^2^ at 0.8 V vs. Ag/AgCl and it delivered 104 µmol of oxygen in 2 h. A very low bandgap of 2.09 eV for this CMP is responsible for achieving high photoelectrochemical activity. Further, the pyrene units in the *π*-conjugated polymer skeleton along with heteroatoms like “N” and “S” and high specific surface area with inherent microporosity are responsible for the commendable photoelectrocatalytic activity and this will open new opportunities in electrochemical/photoelectrochemical water splitting reactions in future.

## Data Availability

The original contributions presented in the study are included in the article/Supplementary Material, further inquiries can be directed to the corresponding author.
