# Supplementary material for: Metal-Free Pyrene-Based Conjugated Microporous Polymer Catalyst Bearing N- and S-Sites for Photoelectrochemical Oxygen Evolution Reaction
Source: Front Chem. 2021 Dec 24;9:803860. doi: 10.3389/fchem.2021.803860 (PMC8739966; doi:10.3389/fchem.2021.803860)
Supplement: Supplementary file 1 [file DataSheet1.docx]

**Supporting Information**

Metal-free Pyrene-based Conjugated Microporous Polymer Catalyst Bearing N- and S-sites for Photo-Electrochemical Oxygen Evolution Reaction

*Sabuj Kanti Das,^1,2^ Sanjib Shyamal,^1^ Manisha Das,^2^ Saptarsi Mondal,^3,4^ Avik Chowdhury,^1^ Debabrata Chakraborty,^1^ Ramendra Sundar Dey^2^ and Asim Bhaumik^1,^**

*^1^School of Material Science, Indian Association for the Cultivation of Science, Jadavpur, Kolkata - 700032, India*

*^2^Institute of Nano Science and Technology, Sector-81, Mohali, 140306, Punjab, India*

*^3^Center for Molecular Spectroscopy and Dynamics, Institute of Basic Science (IBS), Seoul 02841, Republic of Korea*

*^4^Department of Chemistry, Korea University, Seoul 02841, Republic of Korea*

**NMR details**

**1. PYTBE**


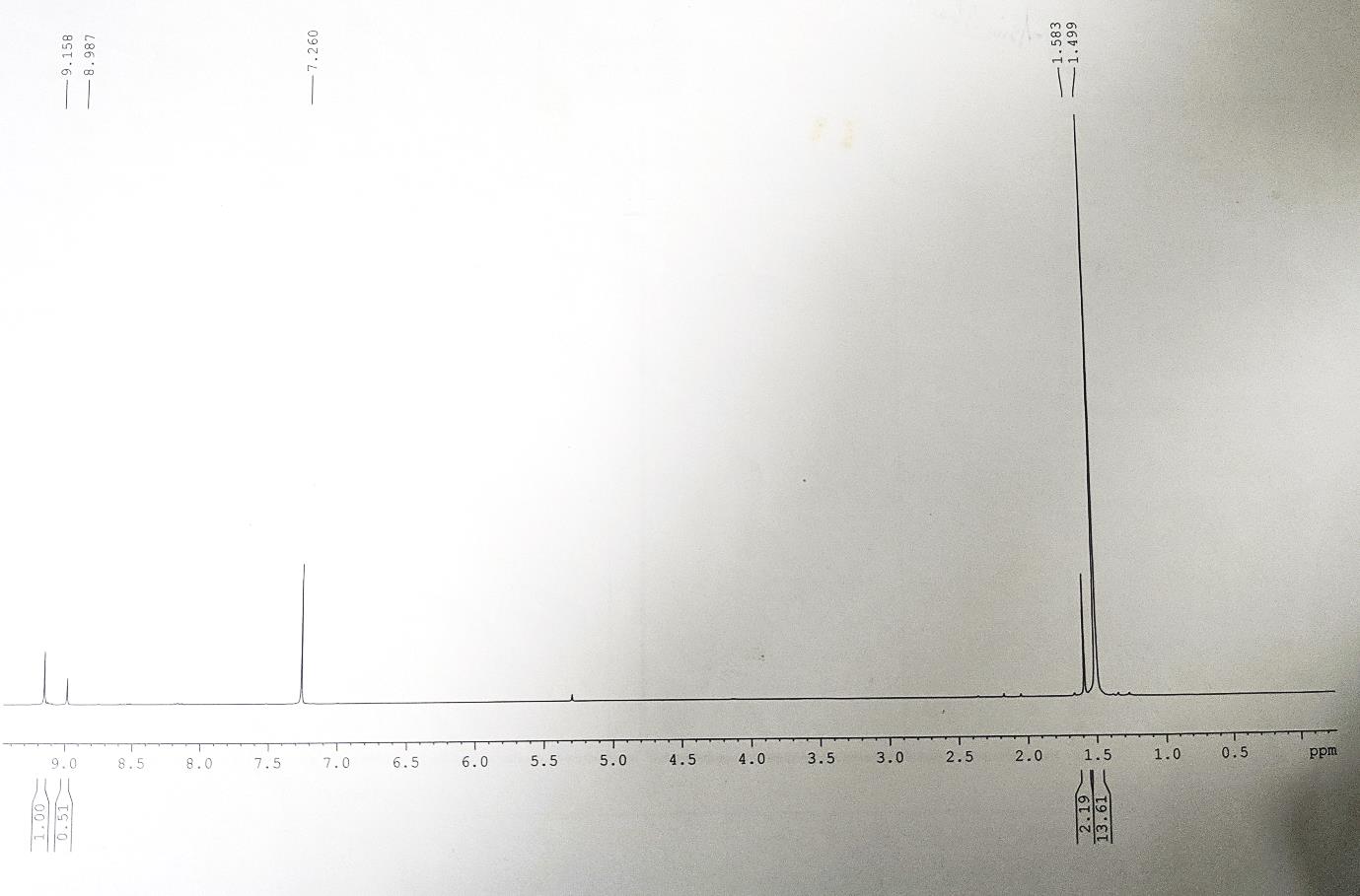
NMR: ^1^H NMR (400 MHz, CDCl_3_, ppm) 9.158 (s, 4H); 8.987 (s, 2H); 1.583 (s, 24H); 1.499 (s, 24H).

**Figure S1**. ^1^H NMR spectrum of PYTBE

**2. DBrBTDZ**


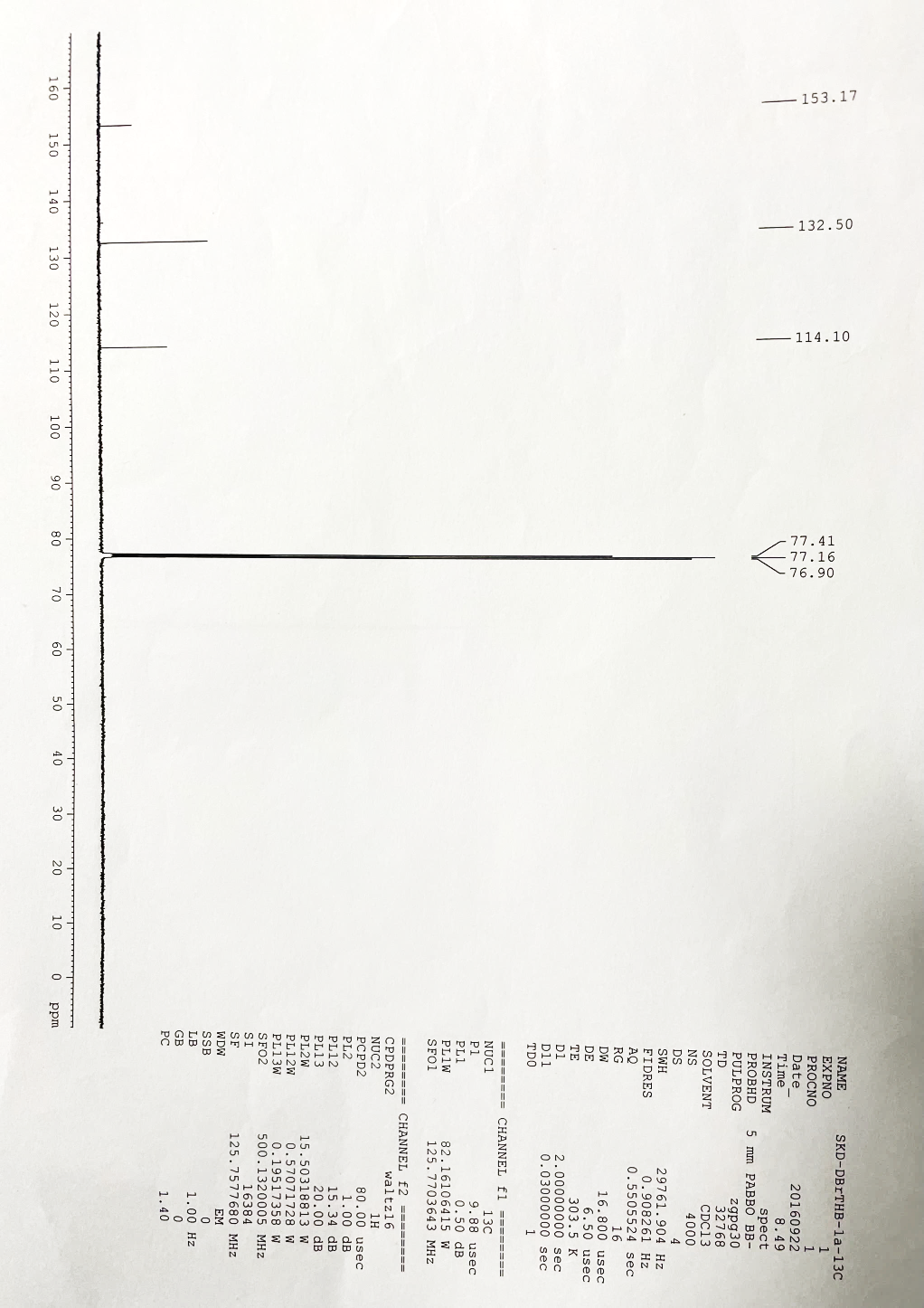
^1^H NMR (500 MHz, CDCl_3_) δ 7.729 (s, 2 H). ^13^C NMR (125 MHz, CDCl_3_) δ 153.17, 132.50, 114.10.

**Figure S2**. ^1^H NMR spectrum of DBrBTDZ

**Figure S5**. ^1^H NMR spectrum of DBrBTDZ


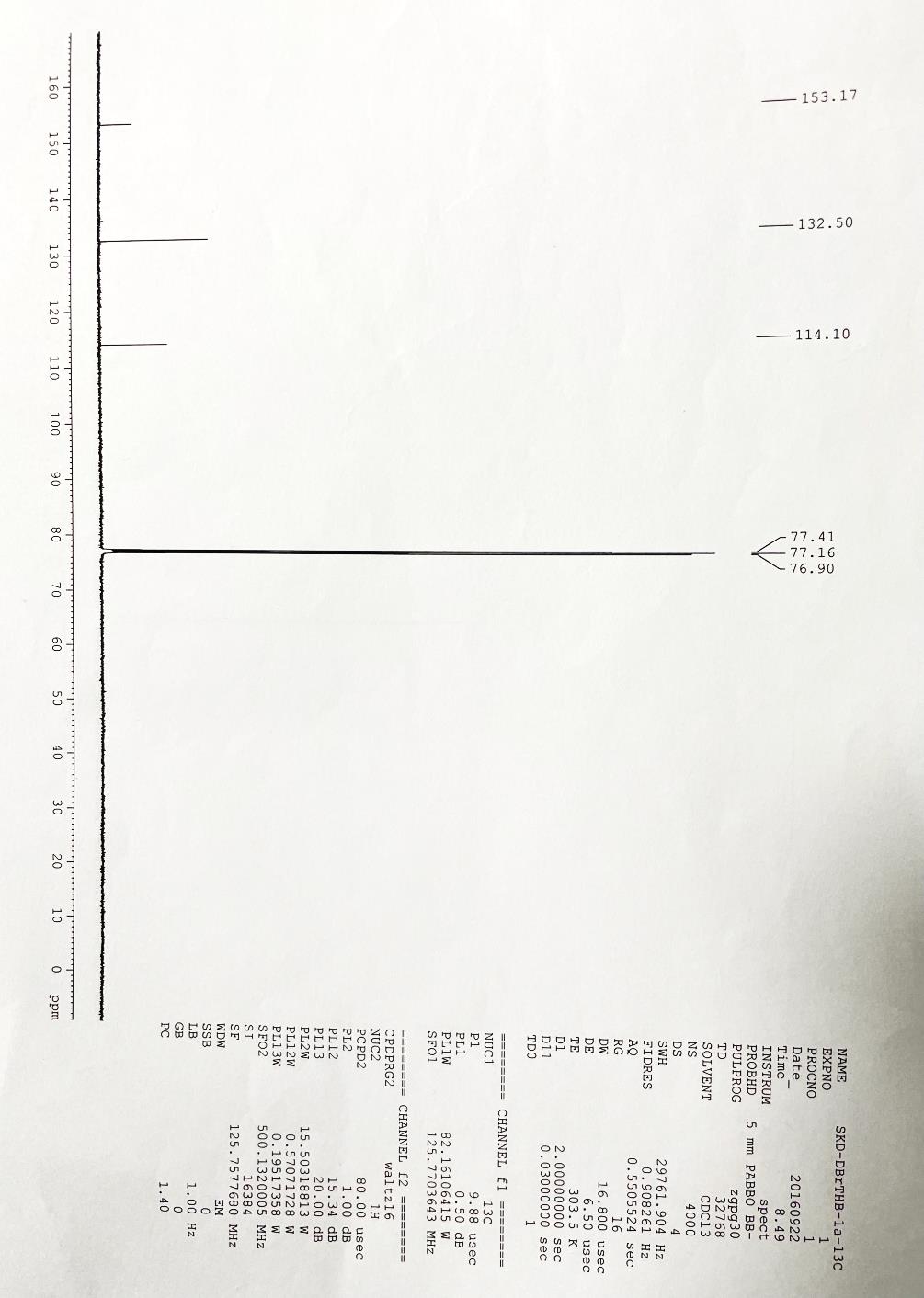


**Figure S3**. ^13^C NMR spectrum of DBrBTDZ.

**Figure S4:** Post photoelectrochemical FTIR spectrum of PBTDZ.

**Figure S5**. PXRD pattern of PBTDZ.

**Figure S6.** Thermogravimetric analysis (TGA) of PBTDZ.

**Figure S7**. N_2_ adsorption/desorption isotherm of base treated PBTDZ. Corresponding pore size distribution is shown in the inset.

**Figure S8**. XPS survey pattern of PBTDZ with C1s, N1s. and S2p spectra


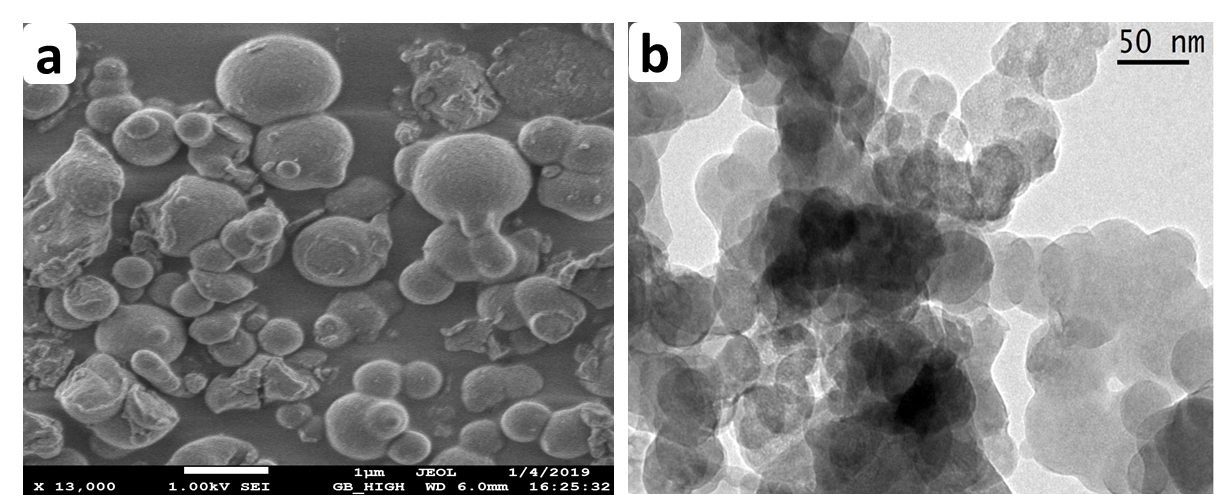


Figure S9. Post catalysis FESEM (a) and HRTEM images of PBTDZ (b).

**Figure S10**. Electrochemical impedance spectra of PBTDZ
